# Morphological and Functional Analysis of Hepatocyte Spheroids Generated on Poly*-*HEMA-Treated Surfaces under the Influence of Fetal Calf Serum and Nonparenchymal Cells

**DOI:** 10.3390/biom3010242

**Published:** 2013-03-07

**Authors:** Ali Acikgöz, Shibashish Giri, Man-Gi Cho, Augustinus Bader

**Affiliations:** 1 Department of Cell Techniques and Applied Stem Cell Biology, Center for Biotechnology and Biomedicine (BBZ), University of Leipzig, Deutscher Platz 5, 04103 Leipzig, Germany; E-Mails: aliacikgoez@yahoo.de (A.A.); augustinus.bader@bbz.uni-leipzig.de (A.B.); 2 Klinikum St Georg, Delitzscher Str. 141, 04129 Leipzig, Germany; 3 Department of Bio-Chemical Engineering, Graduate School, Dongseo University, Busan 617-716, Republic of Korea; E-Mail: mgcho@gdsu.dongseo.ac.kr

**Keywords:** diazepam, fetal calf serum, poly-HEMA, rat hepatocyte, nonparencymal cells, sandwich model, spheroid model

## Abstract

Poly (2-hydroxyethyl methacrylate) (HEMA) has been used as a clinical material, in the form of a soft hydrogel, for various surgical procedures, including endovascular surgery of liver. It is a clear liquid compound and, as a soft, flexible, water-absorbing material, has been used to make soft contact lenses from small, concave, spinning molds. Primary rat hepatocyte spheroids were created on a poly-HEMA-coated surface with the intention of inducing hepatic tissue formation and improving liver functions. We investigated spheroid formation of primary adult rat hepatocyte cells and characterized hepatic-specific functions under the special influence of fetal calf serum (FCS) and nonparencymal cells (NPC) up to six days in different culture systems (e.g., hepatocytes + FCS, hepatocytes – FCS, NPC + FCS, NPC – FCS, co-culture + FCS, co-culture – FCS) in both the spheroid model and sandwich model. Immunohistologically, we detected gap junctions, Ito cell/Kupffer cells, sinusoidal endothelial cells and an extracellular matrix in the spheroid model. FCS has no positive effect in the sandwich model, but has a negative effect in the spheroid model on albumin production, and no influence in urea production in either model. We found more cell viability in smaller diameter spheroids than larger ones by using the apoptosis test. Furthermore, there is no positive influence of the serum or NPC on spheroid formation, suggesting that it may only depend on the physical condition of the culture system. Since the sandwich culture has been considered a “gold standard” *in vitro* culture model, the hepatocyte spheroids generated on the poly-HEMA-coated surface were compared with those in the sandwich model. Major liver-specific functions, such as albumin secretion and urea synthesis, were evaluated in both the spheroid and sandwich model. The synthesis performance in the spheroid compared to the sandwich culture increases approximately by a factor of 1.5. Disintegration of plasma membranes in both models was measured by lactate dehydrogenase (LDH) release in both models. Additionally, diazepam was used as a substrate in drug metabolism studies to characterize the differences in the biotransformation potential with metabolite profiles in both models. It showed that the diazepam metabolism activities in the spheroid model is about 10-fold lower than the sandwich model. The poly-HEMA-based hepatocyte spheroid is a promising new platform towards hepatic tissue engineering leading to *in vitro* hepatic tissue formation.

## 1. Introduction

In the past few decades, hepatic tissue engineering has focused on the improvement of normal hepatocyte cell culture models to develop an organ-specific multicellular cell culture model to restore the stability of the adult hepatocyte’s functions *in vitro* for pharmacological research and hepatocyte research, including bioartificial liver supports. Primary hepatocyte cells are always preferable, as these cells closely mimic the in vivo state and generate more physiologically relevant data than cell lines. *In vitro* culture of primary hepatocytes is a useful model for the expression and regulation of liver genes [1]. However, the main disadvantage is that primary cells lose their state of metabolic function in the conventional monolayer due to the lack of a proper multicellular three-dimensional microenvironment like *in vivo*. To overcome this situation, researchers have developed two widely accepted advanced cellular culture models, such as the sandwich and spheroid models, to restore the metabolic functions over extended periods of long-term cultures, with further stimulation by adding suitable growth factors and using advanced media. Both models have been widely used to study the vast range of basic and clinical research and provide a broad spectrum of liver-specific functions.

The creation of multicellular spheroids with enhanced hepatic functions is an agreeable attempt to mimic the *in vivo* polarity of liver architecture. Under some circumstances, unattached hepatocytes generally do self-assemble into multicellular spheroids. Mature hepatocyte spheroid culture models are similar to a 3D culture model with improved cell–cell and cell–matrix interactions; they also display higher levels of liver-specific functions, such as high cytochrome P450 activity [2], albumin production [3,4,5,6,7,8], long-term culture up to 60 days transferrin secretion [8], ureagenesis [6], and tyrosine aminotransferase induction [3], than are displayed in monolayer cultures. Such a 3D culture model has occurred to recapitulate many in vivo tissue structures and functions [3,9]. Very few hepatocyte spheroid models were established using: a poly-(L-lactic acid ) polymer [10], rock techniques [11], micro-rotation flows [12], alginate scaffolds [13], RGD and galactose-conjugated membranes [14], positive-charged substrates [4], micropatterning techniques [15], nanopillar sheets [16], galactosylated nanofiber scaffold [17], or polyurethane forms [18]. However, hepatocyte spheroids under the influence of fetal calf serum and nonparechyalmal cells have not yet been established. Since 3D polarity is a vital and typical property of hepatocytes *in vivo* and necessary for proper hepatic functions, this present study attempted to create a multicellular spheroid on a poly-(HEMA)-treated surface under influence of fetal calf serum and nonparechyalmal cells.

Sandwich-cultured hepatocytes are a promising cellular model [19]. In our previous study, the rates of metabolite formation are much lower in conventional primary hepatocyte culture models than in the organotypical model [20]. The sandwich culture model enables the conservation of liver-specific characteristics such as cuboidal morphology of hepatocytes, bile canaliculi, tight junctions, and gap junctions [21,22,23,24,25,26]. Furthermore, we recently reported on two compartment models of biotransformation of the drug diazepam in primary human hepatocytes to show that the metabolites of diazepam are present in two compartments (collagen matrix and supernatant with drug–drug interaction in an organotypical model [27]. However, the sandwich model is a well-accepted model for wide varieties of hepatic tissue engineering, including bioartificial liver devices [28,29], toxicology studies [30]. Lee *et al.* [30] recently report that the hepatocyte spheroid-based BAL system may be a noble nominee for treatment of liver failure patients. 

Furthermore, isolated hepatocytes are not able to maintain the cell membrane polarity [31] whereas hepatocytes in spheroids have the ability to repolarize in culture and lead to bile canaculi formation [32,33] and enhanced cytochrome P 450 activities [34,35]. It was recently reported [11] that biochemical activity is superior in the spheroid model to the monolayer of hepatocytes culture on a tissue culture dish and even a collagen-coated dish. Hepatocyte cultures in the spheroid model exhibit enhanced hepatic-specific activities and prolong viability over the monolayer culture [4,8]. Spheroids are self-aggregated three-dimensional structures that are formed when isolated hepatocytes are cultured on moderately adhesive surfaces or in suspension. Such aggregation facilitates the cell–cell interaction, prevents dedifferentiation, and enhances hepatic-specific functions such as albumin secretion [3], transferrin secretion [8], urea synthesis, ammonium metabolism, gluconeogenesis [36], tyrosine amino transferase induction [3], cytochrome P450 1A1 induction, lidocaine metabolism by cytochrome P450 3A2 [8], bilirubin glurioride conjugation activity [38], and a prolonged differentiated state [8]. Hepatocyte cells in spheroids appear to mimic the morphology and ultrastructure as in native liver lobules [5,38,39]. 

In addition, the role of FCS in an *in vitro* culture model is also a pivotal parameter to investigate the effects of FCS on cell culture. Little is known about the effect of FCS on either the sandwich model or the multicellular spheroid model of hepatocytes. Although fetal calf serum (FCS) is a supplement of many cell culture media, providing many necessary growth factors and cytokines for successful culture, many substances present in FCS have not yet been clearly defined, nor are the functions of the cultured cells [40] and the exact effect of serum on the liver cell culture always understood [41]. Wessman and Levings, [42] reported that as much as 20–50% of commercial fetal bovine serum was virus-positive. Hence, FCS may interfere with the experimental outcomes. To answer this question, we aimed to investigate the effect of FCS by experimenting with and without FCS in both the sandwich and spheroid models.

In the present study, we investigated how the spheroid formation depends upon the diameter, the cell concentration of the spheroid, and the effect of FCS in the spheroid formation of adult hepatocyte cultures. We detected various other liver cells (ito cell, Kuffer cell, endothelial cell, bile duct) in the spheroid model. We used here a co-culture with NPS in the proportion found *in vivo* and cultures with and without FCS up to six days (e.g., hepatocyte + FCS, hepatocyte – FCS, NPC + FCS, NPC – FCS, co-culture + FCS, co-culture – FCS). We thus evaluated liver-specific functions (LDH activity, albumin secretion, urea production, HPLC analysis of diazepam of the spheroid model) and compared them to the same conditions in the sandwich model in order to discover which are more suitable.

## 2. Results and Discussion

### 2.1. Role of FCS in the Spheroid Model and Effect of the Spheroid Diameter Size in Monoculture and Co-Culture in the Spheroid Model

**Figure 1:**
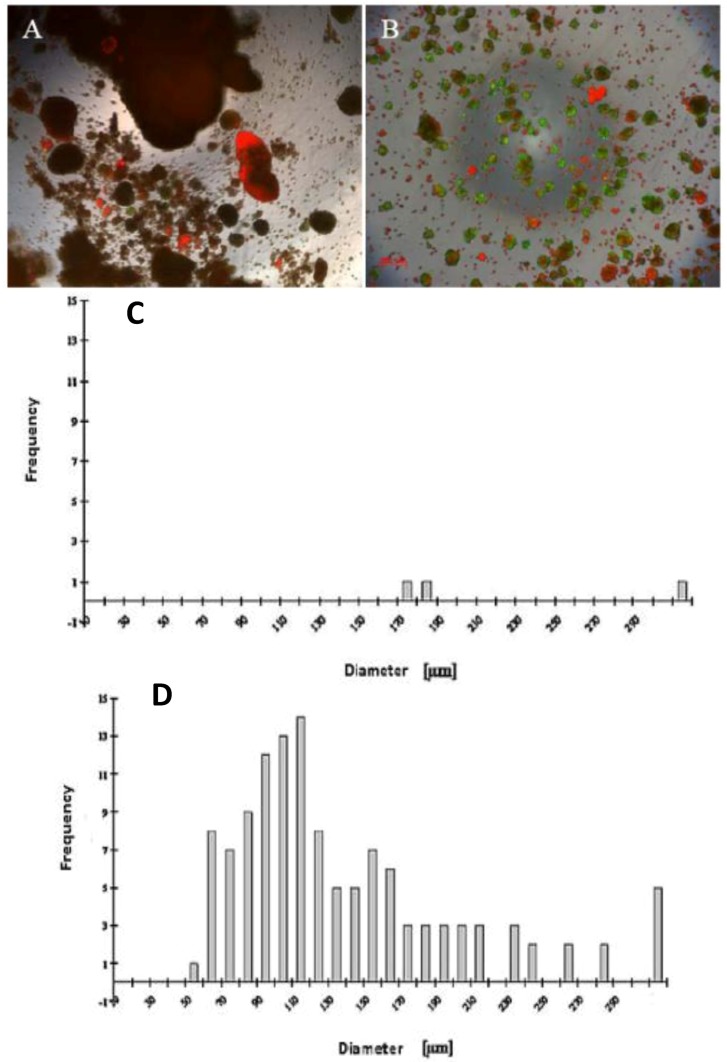
Live/dead test of hepatic spheroid culture in poly-HEMA-treated surfaces. 1 A and B show the live/dead staining of hepatic spheroid culture in poly*-*HEMA-treated surfaces on Petri dishes on the fifth day of culture, Live Cell: green, dead cells: red (A) spheroid with 5% FCS, (B) without FCS (magnification 50×). Scale bar is 100 μm. Figure 1C,D show the frequency distribution of Spheroid diameter on fifth day of culture, hepatocytes (250,000 cells/mL) (C) with 5% FCS or (D) serum-free culture. The diameters of hepatocyte spheroids were measured using a Windows computer with computer-assisted image analyzer. The diameter of spheroids was calculated by converting the spheroid area into an equivalent circle diameter.

Our findings reveal that FCS has a negative influence on the mortality of hepatocytes in the spheroid model (Figure 1A,B), likely due tothe presence of FCS, which contained some substances that are probably not influenced for spheroid formation. We investigated the live/dead staining in the spheroid culture in the Petri dish on the fifth day. There are more dead cells in the culture with 5% FCS, and more live cells in the culture without FCS (Figure 1A,B).

**Figure 2:**
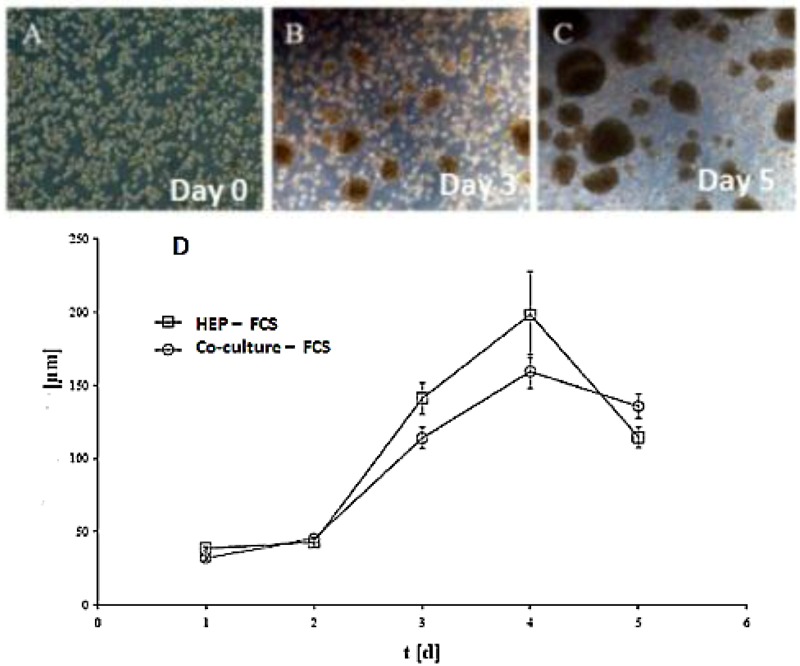
Spheroid formation in serum free culture. Spheroid in serum-free co-culture on day 0 (A); day 3 (B) and day 5 (C); (Magnification: 100×). Scale bar is 100μm. Average diameter of the spheroids in the period of five days in a culture serum-free culture of spheroid. (D) Monoculture from serum-free hepatocytes and a co-culture of hepatocytes (Hep) and NPC.

A wide range of cell numbers of adult hepatocyte spheroids were prepared from 50,000 cells/mL to 70,0000 cells/mL and found the best number in the range of 250,000 cells per mL. The influence of 5% FCS in the spheroid culture shaken in the Petri dish has a negative response. After two or three days of culture cell, clumps occur, finally leading to death due to the presence of FCS. In contrast to the serum-free culture, the spheroid formation took place within 24 to 48 hours of culture. A small number of large cell lumps present in the serum contained culture medium only, while the cultures in the serum culture conditions produce a large number of spheroids without death cell clumps. This result shows that the use of FCS is not necessary for spheroid formation. We analyzed the speed and the size of spheroid formation in both the serum-free and serum-containing culture conditions. Also in this second case, FCS has a negative influence on spheroid formation. We obtained various sizes (30 μm to 290 μm) of spheroids and found a higher frequency of spheroid formation in the size of 110 μm in diameter in only the serum-free culture (Figure 1C,D). Furthermore, we tested the effect of NPC cells in spheroid formation on an average diameter of spheroids up to six days of culture in two approaches: serum-free in a monolayer culture, and in a co-culture of hepatocytes with NPC cells. There are no differences in spheroid formation in both conditions, suggesting that a possible conditioning of the medium with soluble factors by NPC cells has no influence on spheroid formation (Figure 2D). The spheroid formation in the serum-free co-culture on the day 0, day 3, day 5 was seen in Figure 2A,B,C.

### 2.2. Localization of Gap Junction, Ito cells, Kupffer cells*,* Bile Duct Epithelial Cells, Distribution of Hepatocyte Cells in the Spheroid Model

**Figure 3:**
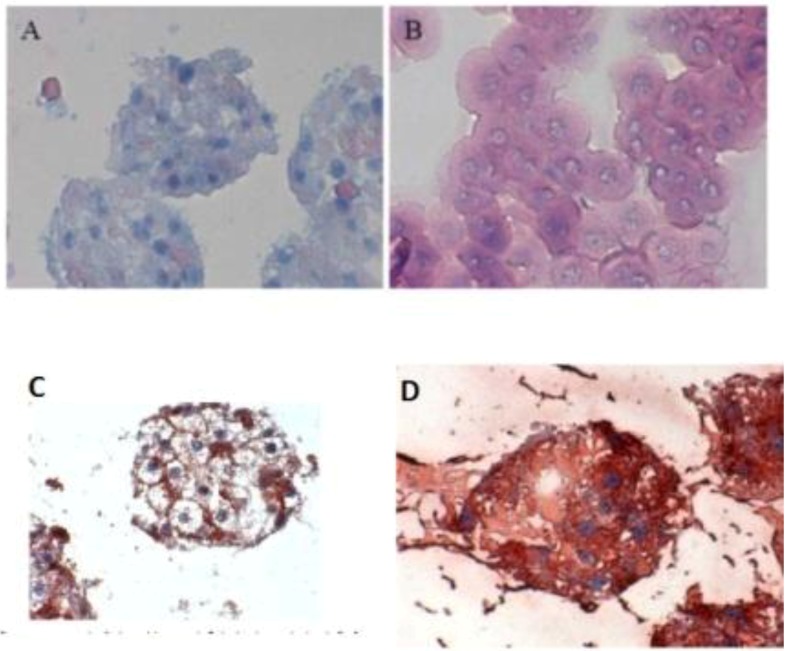
Immunological detection of hepatospecific marker in spheroid culture model (A and B) Extracellular matrix in the spheroid (Magnification 400×), (B) individual cells (magnification 400×). (C) Localization of the gap junctions in Spheroid after incubation with anti-Cx32; (Magnification 400×). (D) Distribution of hepatocytes in the Spheroid (magnification 400×). Scale bar is 100 μm.

When the C 32 antibody was incubated in the co-culture period in the spheroid model, gap junctions were observed. The cell nuclei are blue, while cell–cell contacts (gap junction) are reddish in color, underlined between the cytoplasm red cell borders (Figure 3C). Furthermore, we detected the extracellular matrix in spheroid culture (Figure 3 A,B). The distribution of hepatocytes in the spheroid was detected, where the nuclei are blue and the cytoplasm hepatocytes are red (Figure 3D). It was observed that hepatocytes cells are well distributed in spheroids. We carried out the localization of the Ito cells (Desmin) and Kupffer cells (CD163) in the spheroid model. 

**Figure 4:**
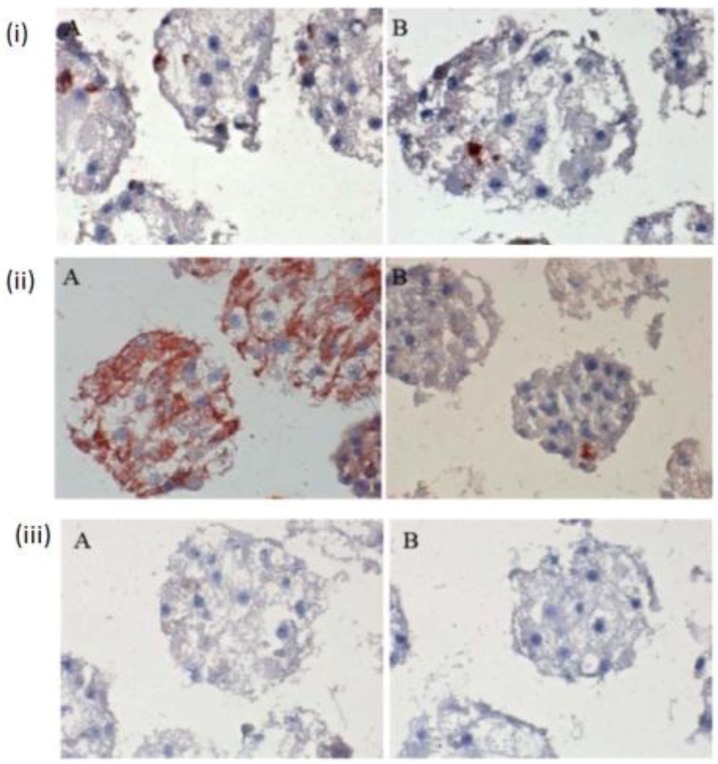
Immunological detection of different liver cells in spheroid culture model **(i) A and B:** localization of Ito cells (A) and Kupffer cells (B) in the Spheroid (magnification 400×). **(ii) A and B:** distribution of the product ductal epithelial cells (A) and endothelial cells (B) in the spheroid; (Magnification 400×), **(iii) A and B:** Negative controls (A) anti-rabbit secondary (B) anti-mouse secondary; (Magnification 200×). Scale bar is 100 μm.

The few existing Ito cells or Kupffer cells are reddish in color, while the nucleus and the cytoplasm are dark blue and light blue in Figure 4(i) A,B,C. These analyses showed how the two cell types are present in the spheroid model. These are only sporadically and arbitrarily distributed in the spheroid model. Furthermore, we found the epithelial cells and bile duct endothelial cells when we incubated with the appropriate antibodies against CD31 and CK18. The biliary epithelial cells are reddish in color, a few sinusoidal endothelial cells are also reddish in color, and the cell nucleus and cytoplasm are dark blue and light blue, respectively, in the hepatocyte spheroid model (Figure 4(ii) A,B,C). In our negative control staining, we did not find any biliary cells, except for cytoplasm and nucleus (Figure 4(iii)A,B,C).

### 2.3. Vitality of Hepatocytes and Apoptotic Test in Different Positions in the Spheroid Model

**Figure 5:**
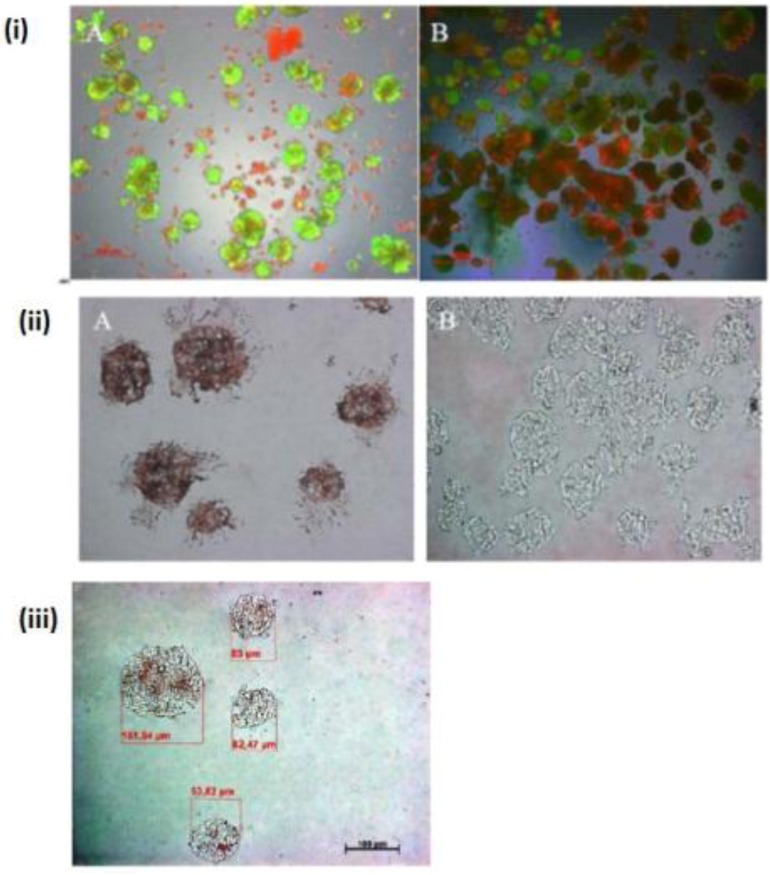
TUNEL staining test in spheroid culture. **(i) A and B** Vitality of cells in the spheroid (A) on day 4 and (B) on day 5 of culture; (Magnification 100×). **(ii) A and B: ** (A) positive control, (B) Negative control of TUNEL staining (magnification 200×), **(iii) A and B:** TUNEL staining of 6-μm sections of spheroids (magnification 200×).

Vitality of cells in the spheroid model is also important. We showed that low vitality of the cells in both individual cells and large spheroids on days 4 and 5 of culture (Figure 5 (i) A,B). It was also observed that dead cells were in irregular cell aggregates (Figure 5 (i) A,B). Based on TUNEL staining in the spheroid culture indicating the apoptotic effect, we showed that the surface cells of the spheroids were full of vital cells, while the cells in the central position had an apoptotic effect. However, according to theoretical calculations, spheroids larger than 100 μm had an apoptotic core. On the other hand, viable cells are present in smaller spheroids. This apoptosis is due to mass limitations (such as O_2_, nutrients) in the interior of the spheroids. These limitations are due to the large removal of the inner cells from the culture medium, and the resulting difficult diffusion of oxygen and nutrients to the cells. The spheroids were in a cross-section of 6-μm cuts, and a cut was subjected to TUNEL staining for the detection of apoptosis in cells (Figure 5 (ii) A,B). The TUNEL staining for 6-μm sections of spheroids confirms shows the results of the aforementioned theory. The red color (apoptosis) in the center of a 6-μm cut spheroid pointed to the apoptotic cells. Parallel to the TUNEL staining, was a positive and a negative control coloring (Figure 5 (iii) A).

The presence of dead cells in the spheroid formation is also possibly due to the lack of the nutrients and oxygen transfer to the inner cells. These apoptotic cells may therefore release lysosomal enzymes into the cell culture medium and damage the other vital cells. We removed the dead spheroids from live spheroids with the isosmotic Percoll solution. This is achieved in an isotonic medium by the separation of live and dead cells that results from their different transporter densities. 

### 2.4. Separation of Dead Spheroids from Live Spheroids, Spheroid Formation

**Figure 6:**
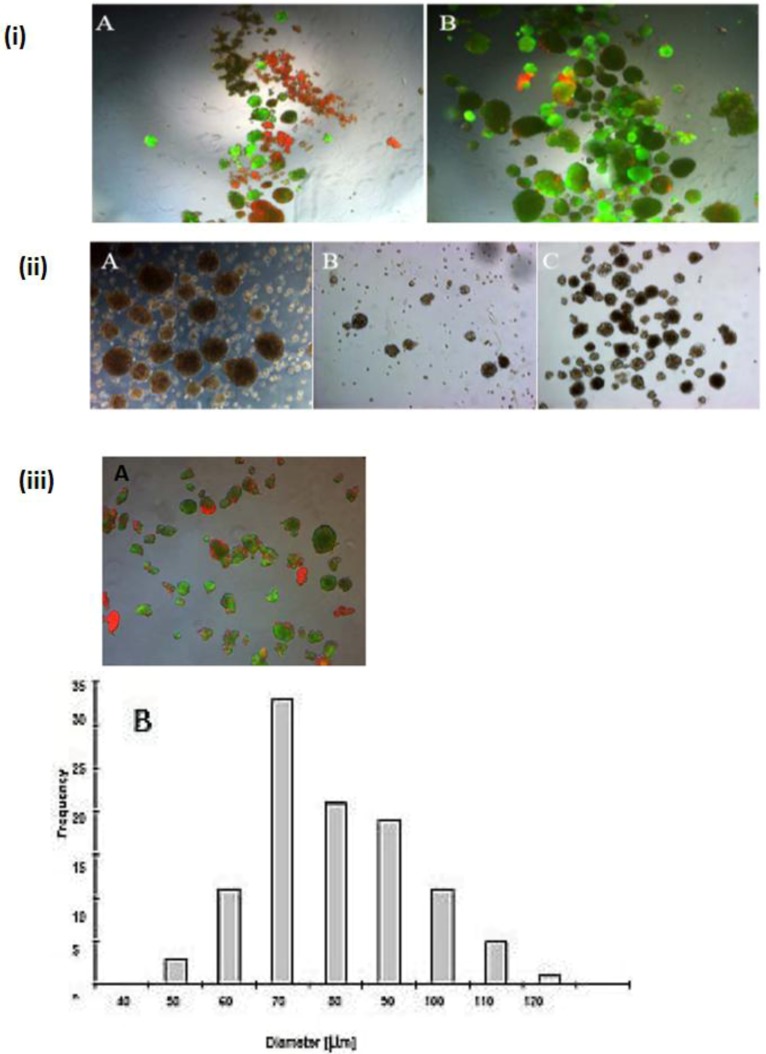
Speriod formation before the Percoll purification and after the Percoll purification. **(i) A and B:** live Tot staining of the cells in the spheroid; serum-free culture (A) before the Percoll purification and (B) after the Percoll purification (magnification 50×). **(ii) A and B:** Serum-free spheroid (A) spheroids before purification, (B) with spheroids Diameter <100 μm (C) spheroids with a diameter of 40–100 μm; (Magnification 100×). **(iii) A and B:** live/dead staining the spheroids after serum-free culture (magnification 100×). Scale bar is 100 μm. (A). Frequency distribution of spheroid diameter after serum-free culture (B).

Dead spheroids were separated from live spheroids. (Figure 6 (i) A,B). It is easy to separate and to remove dead spheroids from live spheroids on a laboratory scale by Percoll purification, but this method is very costly on a larger scale. It is therefore essential to develop a method for fewer dead cells in a spheroid model which avoids this Percoll purification. To overcome this limitation, we tested spheroids of different sizes (more than 100 μm) and diameter (40–100 μm) in order to know the appropriate size of spheroids in which very few cells exist. Finally, we showed spheroids with a diameter of 40–100 μm which had fewer dead cells (Figure 6 (ii) A,B; Figure 6 (iii) A,B,C). We tested the frequency distribution of the spheroid diameter 40 μm to 120 μm and the average spheroid diameter was 75 μm ± 30 μm. (Figure 6 (iii) B) in serum-free culture. We showed that spheroid necrosis occurred inside with increasing diameter size, which was probably due to the limitation of oxygen and nutrients. The method for serum is less effective than the Percoll purification, but it is sufficient. In contrast to the Percoll purification, this method offers a cheaper alternative method for the larger scale. 

### 2.5. LDH Activity in Monoculture and Co-Culture in the Same Culture Conditions in Both the Spheroid and Sandwich Models

**Figure 7:**
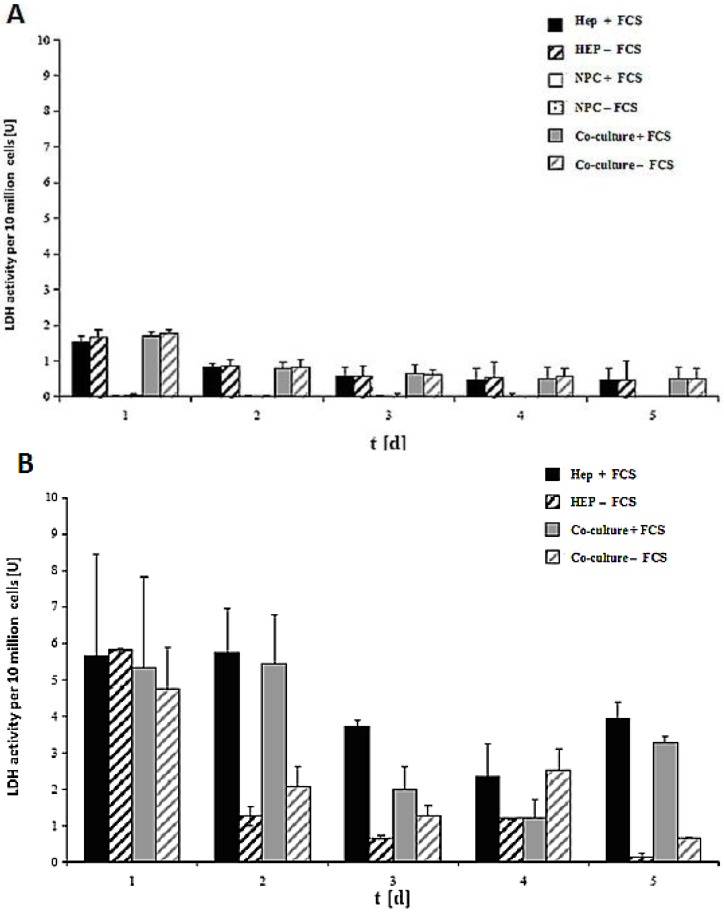
LDH activity of the cells in the sandwich and spheroid culture. **(A):** LDH activity of the cells in the sandwich culture per 10 million from day 1 to day 5. (B): LDH activity of the cells in the spheroid culture per 10 million cells, day 1 to day 5. Each point represents the mean ± S.E.M. of five experiments during which LDH activities were measured in triplicate.

LDH activity of the cell culture of 10 million cells in the spheroid model and sandwich model up to five days is shown in Figure 7A,B. The damage of the cell membrane is higher in serum-containing cultures than in serum-free cultures in both spheroid models, thus suggesting the necessity of avoiding FCS in hepatocyte spheroid cultures, in order to ensure membrane stability (Figure 7A). These experiments showed that FCS retained negative influence on the cells in the spheroids. No significant differences in LDH release are detected between the monoculture from hepatocytes and the co-culture of hepatocytes and NPC (Figure 7B). The comparison of the two models showed that the levels of LDH activity in both systems greatly differed from each other (Figure 7A,B). The maximum values for the LDH activity increased approximately twofold in the sandwich model, and around six-fold in the spheroid model. This figure showed increased stress for the cells in a suspension culture in the spheroid model, as opposed to the sandwich culture. The embedding of the cells between two layers of collagen and thus the formation of the extracellular matrix had a positive impact for the survival of cells in the sandwich model. In both models, there is a reduction in the initial high values in the course of the culture. The high LDH levels in the medium on culture day 2 are due to isolation stress. However, the cells in the sandwich culture of the entire culture time were vital, which has much less LDH activity. This is a positive point for the sandwich culture to maintain the high viability of cells. LDH activity is less in co-culture FCS than in other cases of Hep + FCS, Hep – FCS, co-culture + FCS in the spheroid model. This result suggested that it is good to avoid FCS because of no such significant role of FCS.

### 2.6. Albumin Synthesis in Monoculture and Co-Culture in the Same Culture Conditions in Both the Spheroid and Sandwich Models

**Figure 8:**
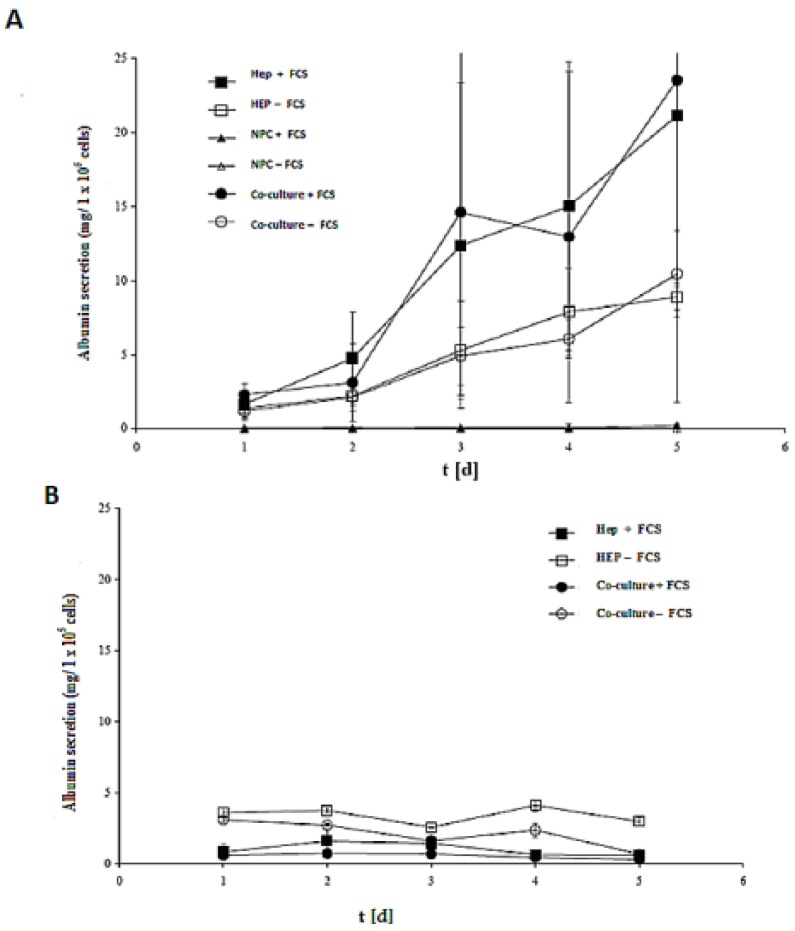
Albumin production in the sandwich and spheroid culture. **(A)** Total albumin production of the cells in serum-free and serum-containing mono-and co-cultures in the sandwich model; Hep + FCS, HEP – FCS, NPC + FCS, NPC – FCS, co-culture + FCS, co-culture – FCS. **(B)** Total albumin production of the cells in serum-free and serum-containing mono-and co-cultures in the spheroid model; Hep + FCS, HEP – FCS, co-culture + FCS, co-culture – FCS. Each point represents the mean ± S.E.M. of five experiments during which LDH activities were measured in triplicate.

The efficacy of both models (spheroid model and sandwich model) was evaluated by assessing the albumin secretion of all cases in the culture time up to five days (Figure 8A,B). Despite the albumin production of the cells in the sandwich model, the LDH activities showed that that the number of live cells of the culture period remained almost constant. In a spheroid model, however, large numbers of cells died (Figure 8A,B). Furthermore, according to the calculation of the total protein content, the yield of living spheroids is only about 10%. On culture day 5, the albumin production is at a maximum in the sandwich model, which is about 25 mg per one million cells, and 4 mg per one million cells in the spheroid model. The synthesis performance in the spheroid compared to the sandwich culture increases approximately by a factor of 1.5.

The role of FCS is a negative one in the spheroid model, yet it has a positive effect in the sandwich model. Hence, our results showed that there is no need for FCS for synthetic performance of albumin secretion, but the opposite is true in the sandwich model. There is no significant difference either in Hep + FCS or co-culture + FCS in both models (spheroid model and sandwich model), thereby indicating that NPC cells have no positive or negative role in albumin synthesis.

### 2.7. Urea Metabolic Activity in the Monoculture and Co-Culture in the Same Culture Conditions in Both the Spheroid and Sandwich Models

**Figure 9:**
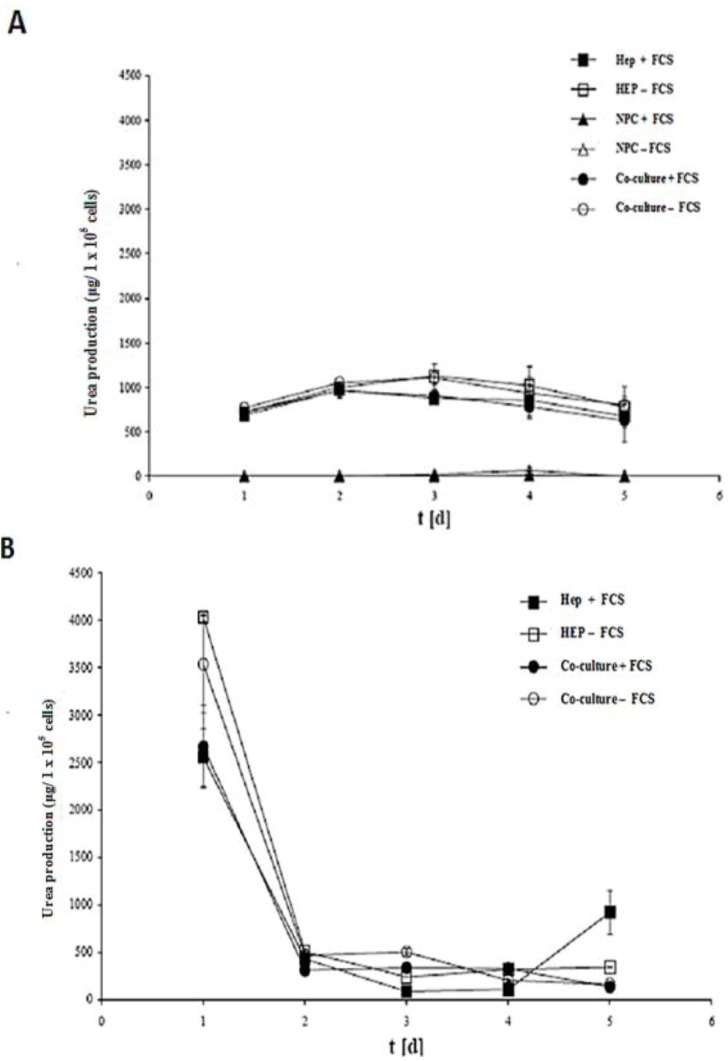
Urea productions in the sandwich and spheroid culture. **(A)** Total urea production of the cells in serum-free and serum-containing mono-and co-cultures in the sandwich model; Hep + FCS, HEP – FCS, NPC + FCS, NPC – FCS, co-culture + FCS, co-culture – FCS. **(B)** Total urea production of the cells in serum-free and serum-containing mono-and co-cultures in the spheroid model; Hep + FCS, HEP – FCS, co-culture + FCS, co-culture – FCS. Each point represents the mean ± S.E.M. of five experiments during which LDH activities were measured in triplicate.

Urea production is another hepatic specific parameter of functional hepatocytes. This parameter is characteristic of their detoxification performance. The urea production of the sandwich and spheroid model in all cases such as hepatocytes + FCS, hepatocytes – FCS, NPC + FCS, NPC – FCS, co- culture + FCS, co-culture – FCS in the spheroid model (Figure 9A) and hepatocytes + FCS, hepatocytes – FCS, co-culture+ FCS, co-culture – FCS in the sandwich model is shown in (Figure 9B). The rate of urea synthesis was 800 μg to 1000 μ g per 1 million cells, which is almost the same in all cases from culture day 1 to culture day 5 in the sandwich model. Urea secretion increased to reach 4000 μg per 1 million cells on day 1 in the monoculture of hepatocytes without serum of the spheroid model and remained at a value of 3600 μg per 1 million cells in co-culture of hepatocyte with NPC in serum-free culture. Interestingly, the urea value dropped in serum plus culture, both in the monoculture of hepatocyte, and the co-culture with NPC in the spheroid model. It indicated that serum has a negative impact on urea metabolism (Figure 9A). When we compare the urea metabolism performance of the spheroid model to the sandwich model, the spheroid is far better than sandwich, as 10% of cells are involved in urea metabolism in the spheroid model, whereas nearly 100% percentages of cells are involved in the sandwich model. NPC has no positive influence on urea metabolism in a monoculture, with or without FCS (Figure 9A). This suggests that the spheroid model, with its special focus on urea metabolism, is not altered by the NPC cell, as it has no positive role. In the sandwich model, the urea production was stable for the entire culture time. In this case also, NPC and FCS have no positive effects on the production of urea. 

### 2.8. HPLC Analysis of Diazepam Metabolites in the Sandwich and Spheroid Models with the Same Culture Conditions

**Figure 10:**
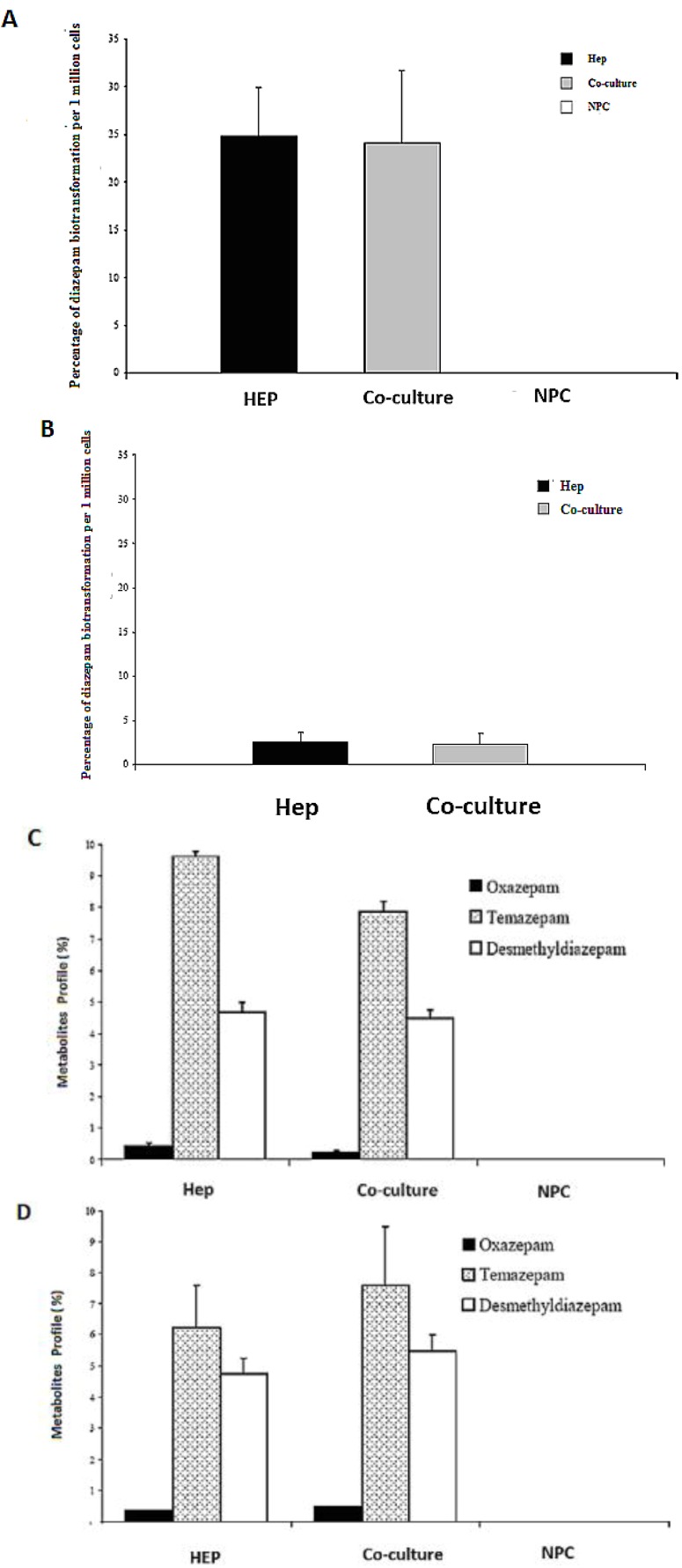
Diazepam metabolism in the sandwich and spheroid culture. **(A)**, Percentage of diazepam metabolism in mono- and co-cultures in the sandwich model; Hep, co-culture and NPC **(B)**, Percentage of diazepam metabolism in mono-and co-cultures in spheroid model; Hep, co-culture and NPC **(C)** Diazepam’s metabolite profile (percentage) in mono- and co-cultures in the sandwich model; Hep, co-culture and NPC, **(D)** Diazepam’s metabolite profile (percentage) inmono and co-cultures in the sandwich model; Hep, co-culture and NPC. Each point represents the mean ± S.E.M. of five experiments during which LDH activities were measured in triplicate.

Diazepam is a very frequently used drug in pharmacotherapy. Diazepam metabolism has been investigated in both sandwich and spheroid models. Percentage metabolites of diazepam in monoculture (hepatocytes) and co-culture (hepatocytes and NPC) in the sandwich model were shown in (Figure 10A). Percentage metabolites of diazepam in serum-free monoculture (hepatocytes) and co-cultures (hepatocytes and NPC) in the spheroid model were shown in (Figure 10B). Percentage of metabolite production of diazepam of the sandwich model (Figure 10 C) and spheroid model is almost the same in serum-free conditions and serum-plus conditions (Figure 10D). Both metabolites (desmethyldiazepam and temazepam) were present in both models, which indicates normal diazepam metabolism. Temazepam concentration was generally higher than desmethyldiazepam concentration. Oxazepam concentration is so low as to be hardly detectable, due to its continuous and quick metabolization in both models. Monoculture of NPC cells has no role in all cases of both models. It implies that NPC has no potential to carry out the biotransformation activities of diazepam. There is a negative effect of NPC cells in a co-culture of the sandwich model, but has the opposite effect in the case of the spheroid model (Figure 10C,D). The percentages of diazepam metabolism activities by the hepatocytes in the monoculture and co-culture for the intermediate metabolite temazepam are 8%, followed by desmethyldiazepam (~5%). In all cases, oxazepam concentration is less than <1%. We showed that the diazepam metabolism activities in the spheroid model is about 10-fold lower than the sandwich model (Figure 10A,B). If the loss is about 90% of the cells in the case of the spheroid model, then it should be considered the same in both models. Taken together, the performance of diazepam metabolism of the spheroid model is better than in the sandwich. 

Spheroid models are a promising tool for both basic and clinical research, as spheroids are multicultural aggregates with a proper geometrical pattern that exhibit a tissue-like morphology. Cell behavior relies on a geometrical pattern of the cellular model, and the spheroid model is better to mimic the *in vivo* geometric pattern. The sandwich model is also an organotypical model which has many advantages, as compared to the conventional model and the spheroid model; it is believed that the spheroid model is superior to the sandwich model from a geometrical point of view. This concept encouraged us to design a comparative study of both models with special references to the effect of NPC and FCS in the same culture conditions.

FCS is routinely added to culture media for maintaining cell culture. FCS is associated with undefined factors which may create problems, such as interference with the experimental outcome, or batch-to-batch variance. Therefore, from a theoretical point of view, FCS is not preferable for cell culture. However, the actual effects of FCS on liver cell culture are not well known [41]. Although there is increased awareness of fetal sensibility to pain, many researchers remain unaware if the fetus receives noxious stimuli during the collection of FCS [43]. The different cell tissue cultures and commercial fetal calf sera (FTS) that have been used in biological and virological research were first screened for detection of the bovine viral diarrhea virus (BVDV, *Pestivirus* genus, Flaviviridae family) and mycoplasma contamination [44]. FCS has been added to cell culture media in both models (sandwich and spheroid model), but little is known about the effect of FCS during the evaluation of the experimental outcomes, either in conventional or advanced cellular models. In the present basis study, we showed that more cells survived in the spheroid model without FCS. FCS is not necessary for spheroid formation, and NPC has no influence on spheroid formation. Hepatocyte cells are able to reassemble the spheroid, and Ito cells, Kupffer cells, or bile duct epithelial cells are well distributed in the spheroid model and gap junctions were found, indicating possible intercellular signaling between them. It has been reported that there is less oxygenation of cells of large spheroids particularly in the central part of the spheroid. We have prepared a wide range of cell numbers and found 250,000 cells per mL provide for a good quality of spheroid formation. Based on our present study, 70 μm of spheroid is suitable in a serum-free culture. 

Generally, the *in vitro* primary cell culture method followed the enzymatic dissociation of the tissue, which destroys cell–cell interactions [45,46]. Conventional culture in culture dishes provides two dimensions where a cell is unable to participate in cell-cell interaction in 3D since it has been demonstrated that cell–cell interaction is critical for functional differentiation [47,48,49]. There is an urgent need for a three-dimensional culture system to facilitate either shorter or longer term cultures of hepatocytes for diagnostics, therapeutic applications, drug evaluation, and basic and clinical screening processes [50]. The spheroid model of the adult hepatocyte culture is a current option that is a widely accepted culture system which provides a three-dimensional arrangement of cells (geometry of real tissue) where cells preserve their original shape and maximize their cell–cell interaction. It is, in fact, the basic requirement for cells to retain their differentiating phenotype through cell–cell interaction, where liver-specific markers such as ferritin, tyrosine amino transferase, a-fetoprotein and albumin are produced at significantly high levels [3,51]. It is very encouraging that hepatocytes cultured in the spheroid model are able to secrete an extracellular matrix involved in the morphogenesis of aggregates [3,51]

Furthermore, liver cells are responsible for approximately 500 biochemical reactions *in vivo*. It is demonstrated that cells taken from tissue and placed two-dimensionally on a culture dish gradually lose the tissue function and die soon thereafter. However, cells in the spheroid are more functional, possibly implying bioartificial devices. There are thus possibilities for the clinical application of the spheroid model. In addition, there are already many studies in the spheroid model, such as those on hepatitis C [52], and used in the bioreactor for bioartificial liver [53] and many other implications for clinical studies. In addition, many scientists encapsulate the primary mature hepatocytes by using various biomaterials like sodium alginate, puraMatrix using this spheroid geometry concept and applied for bioartificial liver supports. It is interesting to know that stem cell researchers also initially follow the concept of the spheroid model and use a hanging drop method to meet the geometrical spheroid shape for differentiation of undifferentiated embryonic cells to insulin producing cells [54]. 

The potential use of adult hepatocytes in a bioartificial liver device requires large quantities of viable and highly active cells. To facilitate the scaling up of the system, liver-specific activities of hepatocytes should be maximized. One way of enhancing the specific activities is to cultivate hepatocytes as multicultural spheroids with a suitable diameter of spheroid for the rapid production of the spheroid and to avoid apoptotic death. Adult hepatocytes have been widely explored as the cell source in liver tissue engineering for cell-based therapy for orthotropic liver transplantation in acute liver failures and for the correction of genetic defects of various enzymatic functions. One of the greatest challenges of this field is to provide compatible culture conditions for the hepatocytes to maintain their structural and functional polarity in the long term, while maintaining liver functions after being isolated from the liver. 

Roberts and Soames [55] demonstrated that hepatocyte culture cells in spheroids retain their ability to respond to peroxisome proliferators for up to 12 d. It was reported that, in the case of stress protein, Hsp72/3 has been shown to be increased in spheroids initially, but returned back to *in vivo* levels after 3 d in culture. Moreover, Hsp25 was maintained at *in vivo* levels up to 6 d in culture [56]. Hence, the spheroid culture mode has many major advantages over other conventional cultures and is expected to be applicable for temporal bioartificial supports or genetic cures, or even other diseases. The further development of spheroid culture techniques, however, is a significant step toward bioartificial liver supports. It has been observed that there is a maximum chance of cell–cell interaction, tight junction, gap junction and bile canaliculi in the spheroid model [5,38]. The gap junction is an important hallmark for maintaining the integral cellular process, including differentiation and growth control that provide direct intercellular communication pathways, thereby allowing rapid exchange of ions, essential messengers (cAMP, ATP, IP_3_ ) and metabolites up to 1 kD in size. Vinken *et al*., [57] beautifully reviewed the role of gap junctions and channels in control of hepatic life cycle functions and considered these to be the “goalkeepers” of liver homeostasis.

Thus, there is a need to design a better liver construct with the unique features of liver tissue morphology, that is, having one or two cell layers bordered by sinusoidal channels to allow conductive flow of the culture medium. Three-dimensional tissue, which relies solely on mass diffusion for nutrient supply and removal of cell secretions, is not sufficient for long-term applications. It is envisioned that the co-culture of hepatocytes with endothelial cells may enable the formation of conductive capillaries in spheroids. Another alternative is to use proliferating hepatocytes that may replace the necrotic cells. In our present study, the main disadvantage of the spheroid model is that a few cells died in thecenter due to the lack of proper oxygenation in the central part of spheroid model. However, it has the potential to enhance hepatic function. It needs further investigation to develop methods so that the survival of cells in all positions of the spheroid will receive proper oxygen. The spheroid model will then become the best model for hepatic tissue engineering. 

Performances of the bioreactors for the bioartificial liver rely on the detoxification process and is one of the major functions of the liver. We noticed that biotransformation activities of two metabolites of diazepam (oxazepam and tamzapam) is more prevalent in the co-culture than monoculture in the spheroid model, but is found to be the opposite in the sandwich model, thereby indicating that the spheroid model is more appropriate because *in vivo* hepatocyte cells are in close contact with NPC where biotransformation of diazepam occurs.

This spheroid model may provide the ideal building units for tissue reconstruction and is expected to be a promising candidate and the main component of artificial liver support. The clinical success of the bioartificial liver (BAL) depends entirely on the metabolically efficient cellular component. This culture system might offer long-term culture with enhanced liver-specific functions.

## 3. Experimental Section

### 3.1. Rat Hepatocyte Isolation and Separation of Nonparencymal Cells

Hepatocytes were harvested from Male Wistar rats (180–250 g), by the modified collagenase perfusion method [46] and separation of the nonparencymal cells from the parencymal cells was performed exactly as described in the methods [58]. Briefly, the resulting initial cell suspension, (containing both hepatocytes and nonparenchymal cells) was filtered for debris. The filtrate (containing parenchymal and nonparenchymal cells) was subjected to differential centrifugation, a method described previously [59]. The nonparenchymal cells were collected from the first two supernatants of the parenchymal–cell centrifugations. Furthermore, to increase the recovery of nonparenchymal cells from the liver, the residue on the 90 µm mesh filter was incubated for 20 min at 8 °C with 0.25% Pronase (which destroys parenchymal cells), and the nonparenchymal cells were collected and washed (twice) by centrifugation at 400 g for 5min. The cells that floated into the top phase were aspirated and subjected to a 30-s and 50-g centrifugation to remove any remaining parenchymal cells. The nonparenchymal cell preparation was collected and washed by two 400g centrifugations. The nonparenchymal cell preparation was completely free from parenchymal cells or parenchymal cell-derived particles, as judged by phase-contrast microscopy [60]. To carry out the experiments, a vitality average of 90% living cells in both cells was determined by trepan blue exclusion. When in the *in vitro* co-culture, the hepatocytes and the NPC cells were in proper ratio, just as they were *in vivo*. 

### 3.2. Hepatocyte Culture in an Adherent Collagen Sandwich Model

The typical cultivation system for primary hepatocytes is the collagen sandwich as described previously [19,61]. Briefly primary rat hepatocytes were always embedded within two layers of collagen in a modification [19] of the method described previously by Hall *et al.* 1982 and Dunn *et al.* 1989. Rat tail collagen was prepared according to the previous method [64]. A final concentration of 1.5 mg/mL collagen was used for coating Petri dishes (60 mm diameter, Greiner, Frickenhausen, Germany). Four hours following seeding and attachment of the cells, the culture medium was removed along with nonadherent cells and a second layer of 1 mL liquid and ice-cold collagen was pipetted on top of the hepatocytes. After gelation of this second matrix layer, a sandwich configuration with two layers of hydrated collagen gel was obtained to simulate the *in vivo* bipolar hepatocellular enclosure within the matrix of the space of Disse. Within 30 min after gelation of the second layer of matrix, the culture medium was applied. The pH of the collagen was adjusted to 7.4 using a 103 Dulbecco’s modified Eagle medium concentrate, which was diluted with the collagen solution at a ratio of 1:10. Culture medium (2 mL) was changed daily, stored at 4 ºC and used for measurement of albumin, LDH activity, and urea synthesis.

### 3.3. Hepatocyte Spheroid Model on poly-HEMA-Treated Surfaces

The method of the spheroid model of rat adult hepatocytes is described elsewhere [8]. The required number of cells was suspended in a living-appropriate medium volume for the suspension culture and placed in a Petri dish. Petri dishes were previously prepared with poly-coated Hema (2-hydroxyethyl methacrylate) to form spheroids and to prevent cells from attaching to the bottom surface. To shake a coating the cells do not generally grow on the piston because of the flow. Due to the different geometry, the system shook on a Plate (Orbit 3 mm) at 150 rpm , while the Petri dishes were shaken on an orbital (orbit 20 mm) at 50 rpm. The cell culture was carried out in a CO_2_ incubator at 37 °C and 5% CO_2_. 

### 3.4. LDH Activity

LDH (lactate dehydrogenase) was quantified with a colorimetric enzymatic test kit (Cytotoxicity Detection Kit, Roche).

### 3.5. Albumin and Urea Analysis

Specific secretion rates were calculated from albumin levels in a 24-h culture supernatant by ELISA as described by Dunn *et al*. [63]. Albumin secretion was determined by enzyme-linked immunosorbent assay (ELISA), using antibodies specific to rats (Organon Teknika, Durham, DC). Chromatographically purified albumin for standards and the monoclonal antibody for albumin were purchased from Cappel (Durham, NC, USA). 96-well plates (Nunc, Wiesbaden, Germany) were coated with 50 mg/L of albumin and stored at 4 ºC overnight. After washing the plates four times, 100 mL of sample were added to the wells and incubated with 100 mL of anti-rat (or antihuman) albumin antibody conjugated with horseradish peroxidase. After 24 h at 4 ºC, staining buffer containing tetramethylbenzidin (Sigma) and H2O2 (Merck) was added for 7 min. The subsequent addition of 50 mL of 8N H2SO4 (Merck) stopped the reaction. The absorbance of standards and samples was measured at 450 nm *versus* 630 nm using a Tecan Spectophometer. Urea secretion rates were calculated from urea levels in a 24-h culture supernatant. Urea was quantified with a blood urea nitrogen kit (Sigma Aldrich, Deisenhofen).

### 3.6 HPLC Analysis of Diazepam Metabolites (*In vitro* Studies for the Assessment of Diazepam Biotransformation in Both Models)

Rat hepatocytes in both models (sandwich model and spheroid model) were exposed to 15μg/mL diazepam on the third day of culture following pre-exposure to a single dose of an inducer. Controls were not exposed to the drug diazepam. For all respective time points, experiments were run in duplicate. All experiments were repeated four times using cells from 4 individual isolations. For each time point, a different Petri dish was harvested: supernatants of 2 mL each were aspirated and frozen separately at 20 °C until HPLC analysis. 1 μg midazolam as internal standard and 20 μL 4 M NaOH (set to pH 8.0) were added to 1 mL of each probe. After adding 100 μL isopropanol, probes were extracted after 30 min with 5 mL ethylacetate and centrifuged 10 min with 200 g. Ethylacetate-phase was evaporated under nitrogen-atmosphere and the remnant was dissolved in 120 μL mobile phase of HPLC consisting of acetonitrile + methanol + 0,04% triethylamine (40 + 10 + 50 volume parts) pH 7. 80 μL were loaded on the HPLC Nucleosil®- 100-5 C18 HD-column from Macherey-Nagel, Germany. The HPLC equipment was from Merck and Hitachi and consisted of the HPLC pump L7100, the autosampler L7200, UV detector L7450, interface module D7000. For the HPLC analysis of diazepam and product concentrations the samples were thawed in the 120 μL solvent and mixed well. Subsequently, the total volume was in HPLC sample tubes. Measurements took place with a reversed-phase (RP) HPLC at a wavelength of 236 nm instead (Column: LiChrospher 100RP-18e). The flow rate of the mobile phase was 0.8 mL min−1, column-temperature was 22 °C. For the quantification of temazepam, desmethyldiazepam and oxazepam standards of 10 to 10 μg were extracted and measured as described above. With the help of internal standards (midazolam) on the basis of the peak areas concentrations of various metabolites were calculated. 

### 3.7. Histological Dyeing Methods (Staining the Spheroids on the Vitality (Live/Death Staining)

To check the vitality of the spheroids in individual samples, a staining method was used (a mixture of calcein AM and ethidium homodimer). Live cells are distinguished by the presence of ubiquitous intracellular esterase activity, determined by the enzymatic conversion of the virtually nonfluorescent cell-permeant calcein AM to the intensely fluorescent calcein. The polyanionic dye calcein is well retained within live cells, producing an intense, uniform green fluorescence in live cells (ex/em ~495 nm/~515 nm). EthD-1 enters cells with damaged membranes and undergoes a 40-fold enhancement of fluorescence upon binding to nucleic acids, thereby producing a bright red fluorescence in dead cells (ex/em ~495 nm/~635 nm). EthD-1 is excluded by the intact plasma membrane of live cells. Then, 100 μL of the sample was taken from the spheroid culture and purified in spheroid suspension and transferred to the above solution. After a careful mix with the pipette, the microplate is centrifuged at 50 × g for 5 minutes, all on spheroids located at the bottom. The plate was incubated for 30–60 minutes at 37 ºC. The cells were monitored microscopically for the presence of the green calcein AM (Ex: 485 nm and Em: 515 nm) or red EthD-1 (Ex: 525 nm and Em: 590 nm) fluorescence. The fluorescence can be quantitatively measured with a fluorescence microplate reader. The size of the spheroid was evaluated by a cross-section of hepatocytes of the spheroid under a light microscope. 

### 3.8. TUNEL Assay

Apoptosis was detected by terminal deoxynucleotide-mediated nick and labeling (TUNEL) assay by [65]. Paraffin-embedded liver tissue was cut into 5-micometer sections. After deparaffing in serial alcohol solutions, a TUNEL kit was used according to the manufacture’s protocol. Positive and negative control was done using sections pre-treated with DNAse 1 (Sigma Co., USA) and Staining without deoxynucleotide substrates, respectively. Finally, apoptotic hepatocytes were calculated.

### 3.9. Immunocytochemistry

The various NPC cells and other cells (gap junction, Ito cell/Kupffer cell, hepatocytes, ductal epithelium, endothelial cell) and extracellular matrix in the spheroid were to be detected by using the immunostaining method. Briefly, at prefixed time points, membrane-bound cells were washed by pre-warmed PBS three times and fixed with cold absolute methanol for 30 min at 20 ºC. After rinsing with PBS, samples were blocked at room temperature for 1 h using the blocking buffer (3% BSA and 0.1% Triton X-100 in PBS). Thereafter, cells were incubated with various mouse-like monoclonal anti-cytokeratin 18 antibody, C 32 gap junction, CD 163 Kupffer cell , Cd endothelial cell, Desmin Ito cell and dilution in PBS (1:500), (1:500), (1:200), (1:1600), (1:200), respectively, at 4 ºC for 24 h in a humidified chamber. After rinsing three times with 0.1% Tween-20 in PBS (PBST), the cells were incubated with a TRITC-conjugated anti-mouse IgG antibody (Sigma, Singapore) at 20 ºC for 30 min, followed by rinsing three times with 0.1% PBST. Finally, samples were visualized under a florescence microscope. 

### 3.10. Statistical Analysis

At least three cultures from each of the three different hepatocyte isolations were evaluated for each treatment. Data are reported as mean standard deviation. Student’s t-tests were used to assess the statistical relevance of all acquired data. Numerical values of probability smaller than 0.05 were considered as statistically significant.

## 4. Conclusions

This establishing of hepatocyte spheroids generated on poly-HEMA-treated surfaces, with its capability of forming hetero-cell aggregates with other liver cells, will offer an efficient experimental avenue for biological research, drug screening and regenerative medicine. Poly-HEMA as an excellent biocompatible polymer has been used for various surgical proposes, such as endovascular occlusion in pediatric surgery, endovascular occlusion of branches of hepatic artery, and endovascular surgery of liver [66,67,68,69]. Functional poly-HEMA-based hepatocyte spheroids may be a good candidate for cell-based therapy for liver patients in the near future. The presence of the different liver cells in our multicellular spheroid culture model, which mimic *in vivo*-like morphology and enhance hepatic functions better than in the sandwich model, provides significant clues that could lead to a new tool for the study of liver cell interactions and functions, and also for the application in a bioartificial liver support system. Furthermore, the spheroid model without FCS is a better culture model than the sandwich culture and has a potential for implication in basic and clinical studies. In the spheroid model, cells can re-assemble into histotypic structures, better mimicking *in vivo* morphology and enhancing hepatic functions than in the sandwich model. Further research for evaluating the effect of different parameters in the spheroid model, such as pH, temperature, and oxygen consumption in various functions, from the gene to tissue level, are recommended. This research will, in turn, open a new vista in the field of hepatic tissue engineering and other future biomedical research. 
